# RhoE Deficiency Produces Postnatal Lethality, Profound Motor Deficits and Neurodevelopmental Delay in Mice

**DOI:** 10.1371/journal.pone.0019236

**Published:** 2011-04-28

**Authors:** Enric Mocholí, Begoña Ballester-Lurbe, Gloria Arqué, Enric Poch, Blanca Peris, Consuelo Guerri, Mara Dierssen, Rosa M. Guasch, José Terrado, Ignacio Pérez-Roger

**Affiliations:** 1 Department of Chemistry, Biochemistry and Molecular Biology, School of Health Sciences, Universidad CEU Cardenal Herrera, Moncada, Valencia, Spain; 2 Department of Animal Medicine and Surgery, School of Veterinary Sciences, Universidad CEU Cardenal Herrera, Moncada, Valencia, Spain; 3 Genes and Disease Program, Center for Genomic Regulation (CRG), Barcelona Biomedical Research Park (PRBB) and CIBER de Enfermedades Raras (CIBERER), Barcelona, Spain; 4 Laboratory of Cellular Pathology, Centro de Investigación Príncipe Felipe, Valencia, Spain; Institut de la Vision, France

## Abstract

Rnd proteins are a subfamily of Rho GTPases involved in the control of actin cytoskeleton dynamics and other cell functions such as motility, proliferation and survival. Unlike other members of the Rho family, Rnd proteins lack GTPase activity and therefore remain constitutively active. We have recently described that RhoE/Rnd3 is expressed in the Central Nervous System and that it has a role in promoting neurite formation. Despite their possible relevance during development, the role of Rnd proteins *in vivo* is not known. To get insight into the *in vivo* function of RhoE we have generated mice lacking RhoE expression by an exon trapping cassette. RhoE null mice (RhoE gt/gt) are smaller at birth, display growth retardation and early postnatal death since only half of RhoE gt/gt mice survive beyond postnatal day (PD) 15 and 100% are dead by PD 29. RhoE gt/gt mice show an abnormal body position with profound motor impairment and impaired performance in most neurobehavioral tests. Null mutant mice are hypoactive, show an immature locomotor pattern and display a significant delay in the appearance of the hindlimb mature responses. Moreover, they perform worse than the control littermates in the wire suspension, vertical climbing and clinging, righting reflex and negative geotaxis tests. Also, RhoE ablation results in a delay of neuromuscular maturation and in a reduction in the number of spinal motor neurons. Finally, RhoE gt/gt mice lack the common peroneal nerve and, consequently, show a complete atrophy of the target muscles. This is the first model to study the *in vivo* functions of a member of the Rnd subfamily of proteins, revealing the important role of Rnd3/RhoE in the normal development and suggesting the possible involvement of this protein in neurological disorders.

## Introduction

Mammalian Rho GTPases comprise a family of intracellular signaling molecules which, by interacting with target proteins, control a variety of cellular functions such as cell adhesion, cell cycle progression, cell migration, cell morphogenesis, gene expression and actin cytoskeleton dynamics [1], [2], [3]. Rnd GTP-binding proteins compose a subgroup of the Rho GTPase family, with unique properties. They have been found only in vertebrates and, while most Rho GTPases switch between an active GTP-bound form and an inactive GDP-bound form, Rnd proteins are always bound to GTP, which suggests alternative regulation mechanisms such as gene expression regulation, posttranslational modifications and/or protein interactions [4], [5], [6].

RhoE/Rnd3 (hereafter referred to as RhoE) is a member of the Rnd subfamily [7], [8], which is also composed by Rnd1 and Rnd2 [4]. RhoE is thought to bind and inhibit the RhoA effector ROCK I [9] and to interact with p190-Rho-GAP increasing the GTPase activity of RhoA and thus, inactivating it [10]. As a consequence, RhoE antagonizes RhoA function altering the actin cytoskeleton organization and inducing cell motility [8]. In addition, RhoE is involved in the control of cell cycle and survival in some cell lines [11], [12], [13], and it also plays a role in the development and function of the Central Nervous System (CNS) [14], [15].

The functions of individual Rho GTPases have been mainly described by expressing constitutively active and dominant negative mutants. In the last years, analysis of mice that lack some of the Rho proteins has provided very valuable information about the role of these small GTPases in developmental processes and cell behavior [2]. Knockout mice studies have even produced different information to what has been predicted from dominant negative mutant analysis, a difference that can be sometimes explained by the functional redundancy between closely related Rho isoforms. Importantly, knockout mice have also provided models for human diseases, as is the case, for instance, of the RhoGAP oligophrenin in mental retardation [16].

The functions of Rho GTPases seem to be especially important in the nervous system since individual Rho proteins have been shown to play important roles in the regulation of neuronal development, survival, and death. This has been considered particularly relevant in the context of specific neurodegenerative disorders in which Rho family GTPase function is altered [17].

While most of the functions of Rnd proteins have been studied *in vitro*, little is known of their roles *in vivo*. A recent work has shown that Rnd2 is essential for brain development [18], suggesting that Rnd proteins could play important roles during development. To study the role of RhoE *in vivo* we have generated mice lacking RhoE expression by an exon trapping cassette. Our results show that RhoE is essential for postnatal development since RhoE null mice die shortly after birth. In addition, the lack of RhoE expression results in important structural defects, such as the absence of the common peroneal nerve, and notable motor and behavioral deficits.

## Results

### RhoE gt/gt mice generation

**Figure 1:**
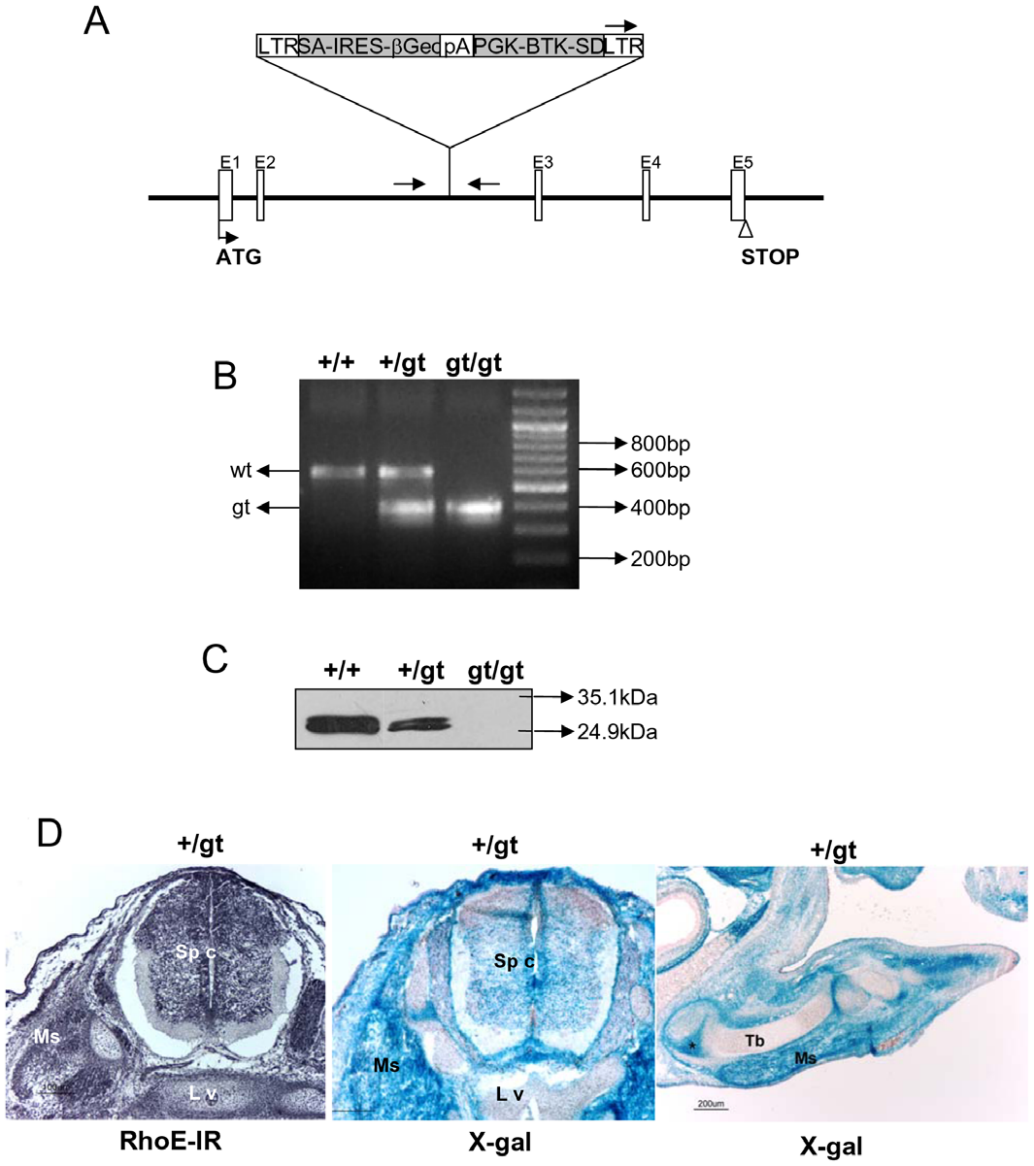
RhoE gene-trap strategy. **A**) The gene trapping cassette in the retroviral vector VICTR37 was found in the second intron of the *RhoE* gene, as assessed by reverse PCR. LTR, viral long terminal repeat; SA, splice acceptor sequence; IRES, internal ribosome entry site; βGeo, fusion of beta-galactosidase and neomycin phosphotransferase genes; pA, polyadenylation sequence; PGK, phosphoglycerate kinase-1 promoter; BTK-SD, Bruton's tyrosine kinase splice donor sequence. **B**) Genotyping was performed as described in Materials and Methods by PCR. The position of the primers used is marked in **A** by arrows. The gene-trap allele (gt) yields a lower molecular weight band than the wild type allele (wt). **C**) Western blot with anti-RhoE antibodies to confirm the absence of RhoE expression in cells from RhoE gt/gt embryos. **D**) Sections showing RhoE immunoreactivity (RhoE-IR, left panel) and X-Gal staining (central and right panels) of a 14.5 dpc RhoE +/gt embryo to reveal expression of the RhoE locus. RhoE-IR is widespread in the embryo with a pattern that matches that of the X-Gal staining. RhoE expression is observed at the lumbar spinal cord (Sp c) and very intense in the striated muscles (Ms), whereas the primordium of the lumbar vertebral body (L v) lacks RhoE-IR (left panel) and X-Gal staining (central panel). A longitudinal section of the hindlimb of the same embryo (right panel) shows high level of X-Gal labeling at the level of the striated muscles and in the joints, whereas the bone primordia lack X-Gal staining. Ms: striated muscles; Tb: tibia primordium. The asterisk marks the knee joint primordium. Scale bar is 100 µm in the left and central panels and 200 µm in the panel on the right.

Heterozygous RhoE +/gt mice were generated by using a gene–trapping method, based on a tagged random mutagenesis, as previously described [19] (**Figure 1A**). Wild type (+/+) and RhoE gene-trap (gt/gt) mice were derived by breeding the heterozygous founder mice on a hybrid genetic background (129SvEvBrd-C57Bl/6J). The insertion of the trapping cassette was confirmed by PCR with DNA extracted from mice tails (**Figure 1B**). We ensured the absence of RhoE expression in gt/gt animals by western blot (**Figure 1C**). Since the trapping cassette has a *lacZ* reporter gene, the RhoE gt homozygous and heterozygous mice have the *lacZ* gene knocked-into the RhoE locus, under the control of the *RhoE* gene regulatory region. Blue staining resulting from the inserted β-galactosidase (β-gal) activity, indicative of *RhoE* gene expression, showed that RhoE was expressed in mouse embryos. X-Gal staining was broadly detected in most embryonic tissues. In addition to other organs, X-Gal staining was appreciable in the central nervous system and in the developing muscles and matched RhoE immunoreactivity (IR) (**Figure 1D**).

### The absence of RhoE expression resulted in growth retardation and postnatal mortality

**Figure 2:**
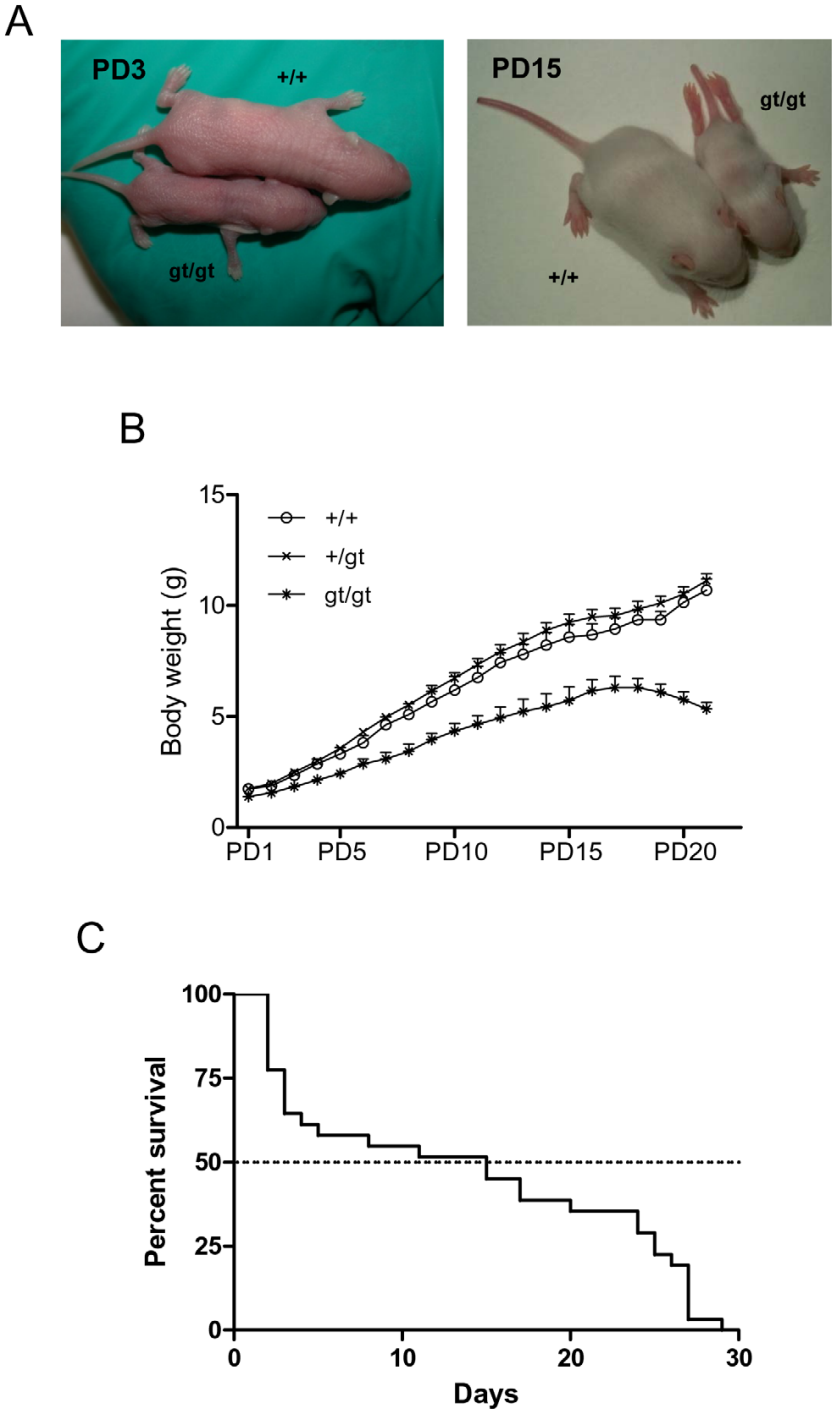
RhoE gt/gt mice are smaller in size and present a limited survival. **A**) Pictures of RhoE gt/gt and +/+ littermates were taken at postnatal day (PD) 3 (left) and PD15 (right) to compare their size. Note the abnormal position of the hindlimbs of the RhoE gt/gt mouse. **B**) All mice (n = 49, 8 wt, 30 +/gt and 11 gt/gt) were weighted everyday from PD1 to PD21. Data are represented as Mean+SD. RhoE gt/gt mice were smaller than RhoE +/+ or +/gt animals at birth (Student's *t* test, p<0.01) and showed a growth delay thereafter (one way ANOVA and Tukey's test, p<0.005). **C**) Kaplan Meier survival curve of a total of 31 RhoE gt/gt mice. All RhoE +/+ and +/gt littermates survived beyond the day when the last RhoE gt/gt mouse died (not shown). The median survival for the RhoE gt/gt mice was 15 days.

To investigate the effect of the lack of RhoE expression on mice viability, we studied the progeny resulting from heterozygous mice crossings. Heterozygous animals were viable and fertile and did not show any apparent abnormality. RhoE gt/gt mice were obtained with a Mendelian distribution (22.3% RhoE gt/gt, 26.4% +/+ and 51.3% +/gt, from a total of 368 newborn mice, p = 0.6791 in a Chi-square analysis when compared to the expected Mendelian frequencies). RhoE gt/gt mice were significantly smaller at birth than their heterozygous or wild type littermates (p<0.01 in a Student's *t* test) and showed a significant growth retardation thereafter (**Figure 2A and 2B** and **Table 1**). The two way ANOVA and Bonferroni posttest indicates that the differences between RhoE gt/gt and the other two genotypes increased from PD1 to PD21 (p<0.0001). Along the pre-weaning period, RhoE gt/gt mice showed a slower daily increase in body weight (p<0.0001 when comparing the slope of the linear regressions), until postnatal day (PD) 18 when they started loosing weight (**Figure 2A and 2B** and **Table 1**); there were no statistically significant differences between the RhoE +/+ and RhoE +/gt groups. The size and weight of the internal organs were reduced but apparently proportional to the body size and weight reduction (not shown). RhoE gt/gt mice did not survive beyond PD29, with a median survival of 15 days (**Figure 2C**), whereas there were no differences in the survival of RhoE +/+ and RhoE +/gt mice (not shown). These results indicate that RhoE expression is necessary for survival as well as for the correct development during the postnatal period.

**Table 1:** RhoE gt/gt mice are smaller than their littermates.

	Weight at birth	Weight at PD18	Daily body-weight increase (slope)	Is the slope different from RhoE +/+?
RhoE +/+	1.74±0.10	9.36±0.46	0.49±0.01	
RhoE +/gt	1.75±0.07	9.84±0.35	0.52±0.02	NO (p = 0.2325)
RhoE gt/gt	1.40±0.05	6.30±0.41	0.32±0.01	YES (p<0.0001)

### RhoE gt/gt mice displayed neurobehavioral development abnormalities

**Figure 3:**
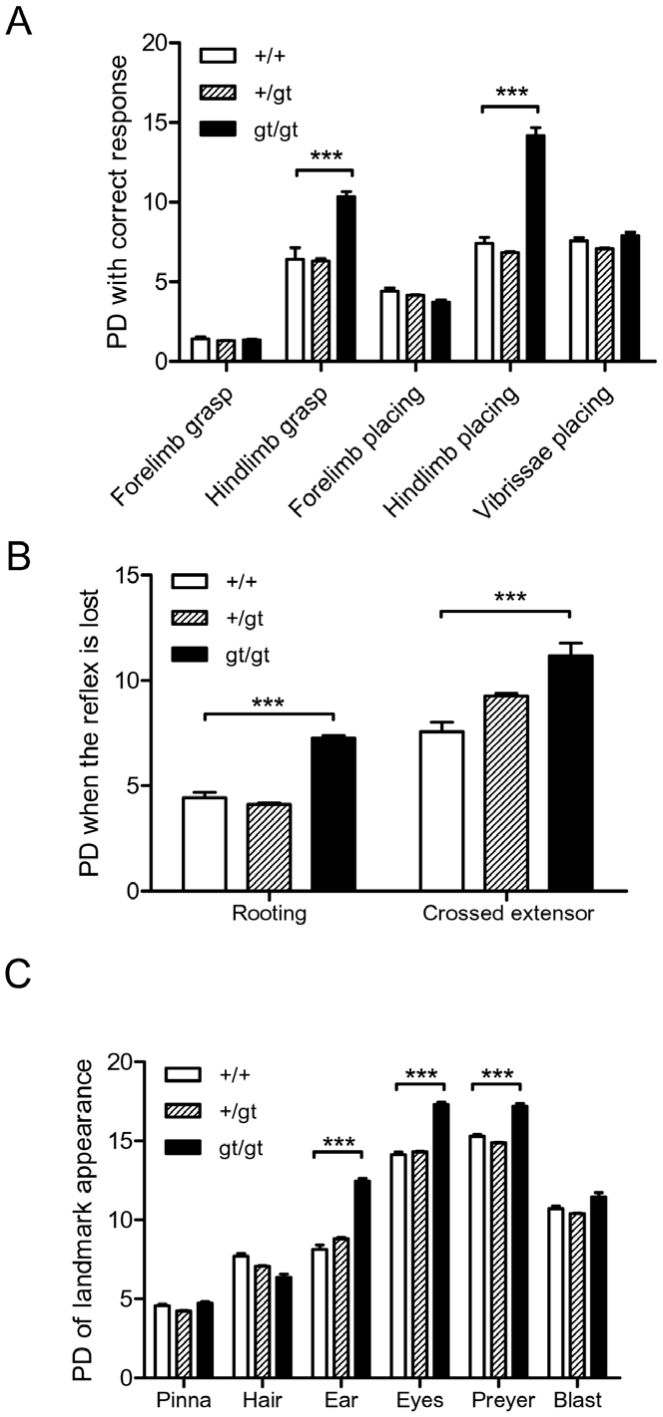
RhoE gt/gt mice displayed neurobehavioral abnormalities. The different reflexes and responses were analyzed as described in Materials and Methods. In all cases the postnatal day (PD) when the response appeared or was lost was recorded. A total of 49 mice (11 RhoE gt/gt, 30 +/gt and 8 +/+) were analyzed. Data are presented as Mean+SEM. Statistically significant differences between RhoE gt/gt and +/+ mice are shown (***p<0.001) **A**) Grasping reflexes and placing responses correlate with the altered hindlimb position in RhoE gt/gt mice. The day when the mice were able to show the correct response was recorded. **B**) Archaic reflexes (rooting and crossed extensor) persist longer in RhoE gt/gt mice than in wt or heterozygous littermates. Columns show the day when the reflex was not observed. **C**) The day when the following development landmarks appeared was recorded: separation of the ears from the head (*Pinna*), apparition of body hair (*Hair*) and opening of the auditive conduct (*Ear*) and of the eyelids (*Eyes*). The delay in the auditory (*Preyer*) and not in the tactile (*Blast*) startle responses is a functional consequence of the delayed ear opening.

In addition to their reduced size, RhoE gt/gt mice also showed some remarkable phenotypic abnormalities that were apparent at birth and whose severity increased with age. These phenotypic anomalies principally consisted in an abnormal body position, affecting mainly the hindlimbs (see **Figure 2A** and **Video S1**). RhoE gt/gt mice are also hypoactive and display general ataxia, spontaneous convulsions and abnormal gait. We performed a set of neurobehavioral tests during the 3 first postnatal weeks to compare the performance of the RhoE gt/gt mice with their wild type littermates. Examination of the sensory and motor reflexes revealed a significant delay (p<0.001) in the appearance of the hindlimb grasping and placing responses (**Figure 3A**). The development of manipulative (grasping, placing) and locomotor abilities of the forelimbs preceded that of the hindlimbs in all genotypes. However, while by PD7 the hindlimb placing and grasp responses could be reliably elicited in wild type and heterozygous mice, they were not found in the hindlimbs until PD10-PD14 in RhoE gt/gt mice. No differences were found between the two groups in the tactile orientation tests such as the vibrissae placing (**Figure 3A**). We found that the archaic reflexes, such as the rooting response and the crossing extensor reflex, disappeared later in the RhoE gt/gt mice than in +/+ or +/gt littermates (p<0.001, **Figure 3B**).

Regarding the emergence of developmental landmarks, both the eyelid opening and the permeation of the auditory conduct took place later in the mutants (p<0.001) and also the functional measure associated with ear opening, the Preyer's reflex, was significantly delayed in the RhoE gt/gt mice (p<0.001, **Figure 3C**). No significant differences among the three groups were observed in the other reflexes and landmarks studied (**Figure 3C**).

### RhoE deficient mice showed severe neuromotor impairment

**Figure 4:**
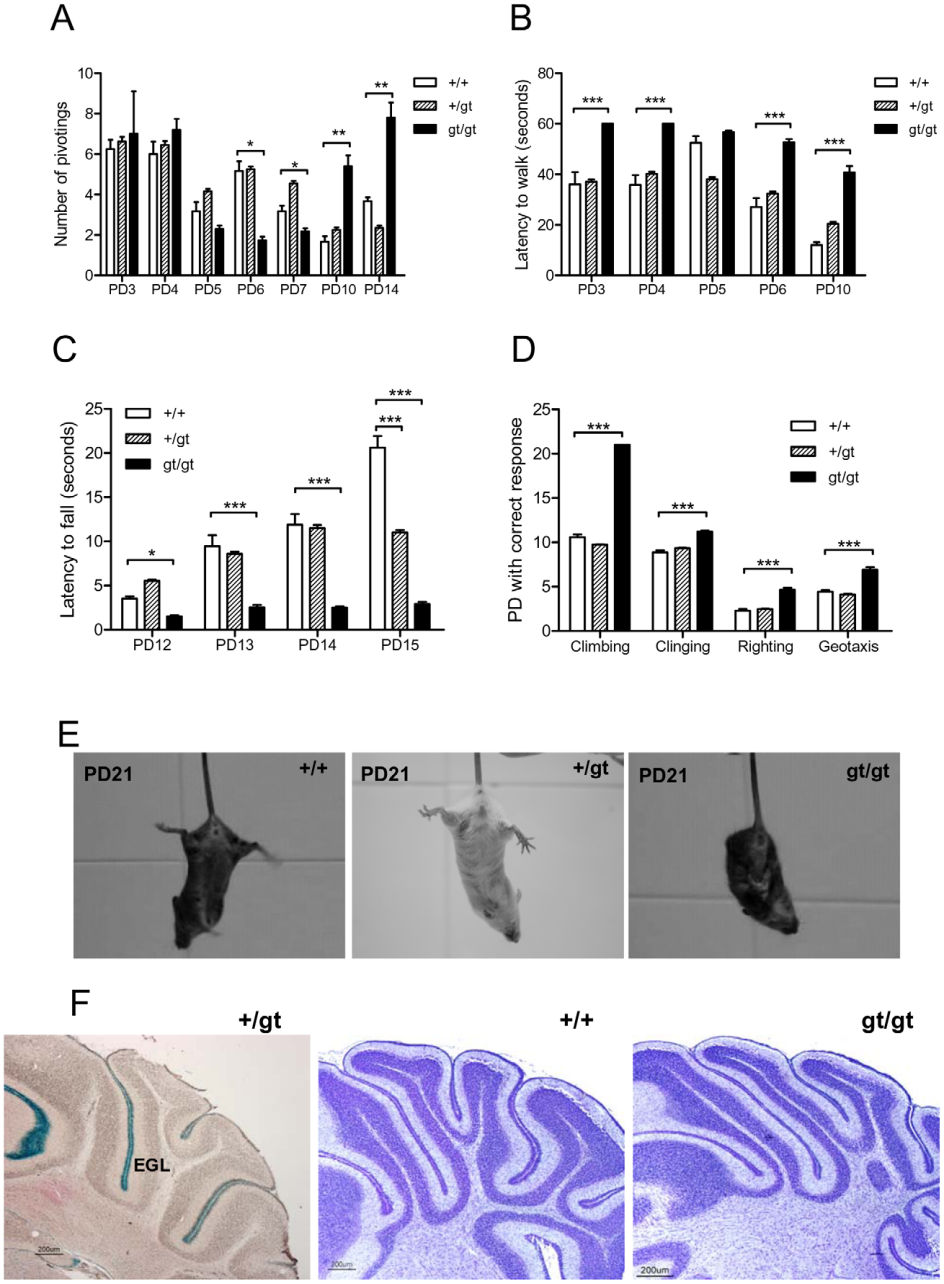
RhoE gt/gt mice show neuromuscular development defects. The same cohort of animals described in **Figure 3** was used. Data are presented as Mean+SEM. Statistical differences between groups are shown when significantly different from the wt controls (*p<0.05; **p<0.01; ***p<0.001). **A**) The number of times that the mouse rotated on its hindlimbs without moving (*Pivoting*) over a 60 second period was recorded at different postnatal days (PD) as indicated. **B**) Latency to walk on a straight line was recorded. At PD 3 and 4, RhoE gt/gt mice showed no walking activity along the maximum time allowed (60 seconds). **C**) In the wire suspension test the latency to fall was significantly reduced in RhoE gt/gt mice. **D**) RhoE gt/gt mice perform worse in different motor tests. The postnatal day (PD) in which the following tests were performed correctly was recorded: vertical climbing (*Climbing*), vertical clinging (*Clinging*), righting reflex (*Righting*) and negative geotaxis (*Geotaxis*). The tests are described in Materials and Methods. **E**) Pictures show an example of the limb clasping response of a PD21 RhoE gt/gt mouse (right) compared with the normal escape posture of a RhoE +/+ (left) and a RhoE +/gt (central) mouse when suspended by the tail. **F**) Sections of P15 cerebella showing X-Gal staining only in the external granular cell layer (EGL, left panel) and the absence of gross abnormalities in the RhoE gt/gt sample (right panel) compared with the wild type (central panel). The sections in the central and right panels are stained with 1% cresyl fast violet solution.

RhoE deficient mice displayed abnormal motor patterns and motor behavior (**Video S1**). To assess the effect of RhoE ablation on the neuromotor development the mouse motor abilities were studied using the pivoting and the walking tests. The immature pivoting locomotion persisted longer in mutant mice than in their littermates (**Figure 4A**). Moreover, the RhoE gt/gt mice tended to be hypoactive especially in intermediate neuromotor development stages (PD 5–7). The persistence of this immature locomotor pattern at later stages (PD 10–14), when straight-line walking appears, indicates a possible delay in the cranio-caudal maturation in RhoE gt/gt mice (**Figure 4A**). This increase in locomotor activity is typical when a gross alteration in the hindlimb activity is present and reflects a compensatory strategy.

The latency to start walking was significantly higher in RhoE gt/gt mice than in the wild types during the first 10 days of life (p<0.001). At PD3 and PD4 mutants were unable to stand on all fours and walk in a straight line in 60 seconds (the maximum time allowed to perform the task), whereas initiation of walking was achieved in less than 40 seconds in the wild types (**Figure 4B**). In the wire suspension test the RhoE gt/gt mice were unable to remain hanging on the wire, being the latency to fall significantly reduced with respect to +/+ and +/gt littermates (p<0.001 at PD13, PD14 and PD15). These differences were more pronounced along postnatal life, with a possible gene-dosage being detected at PD15 (p<0.001 when comparing RhoE +/gt with +/+, **Figure 4C**). In addition, RhoE gt/gt mice presented a worse performance than the controls in the other motor tests assayed, vertical climbing and clinging, righting reflex and negative geotaxis (p<0.001, **Figure 4D**).

To compare the sensorimotor performance of RhoE gt/gt mice with +/+ littermates, we performed the limb clasping test. Limb clasping is a phenotype observed when the animal is suspended by the tail and clenches its limbs instead of showing the normal escape posture. RhoE gt/gt mice displayed a pronounced limb clasping behavior that was absent in +/+ and +/gt mice (**Figure 4E**).

However, the expression of RhoE in the cerebellum at PD15 was detected only in the external granule cell layer, as assessed by X-Gal staining (**Figure 4F**, left panel), reproducing the RhoE-IR previously reported [14]. The cells of this layer proliferate during the first days of postnatal development and later migrate inwardly to form the internal granule cells. At PD15 almost all the cells of the external granule cell layer have already migrated, and only a few cells, which will soon disappear, remain located in the outermost cerebellar cortex. As a consequence, none of the mature neurons in the cerebellar cortex expressed RhoE and no gross histological alteration in the cerebellum of the RhoE gt/gt mice (**Figure 4F**, right panel) compared to the wild type (**Figure 4F**, central panel) was detected.

In summary, all the results shown above suggest that the absence of RhoE expression produces a delay in the neurobehavioral and neuromotor development of mice.

### Neuromuscular alterations and absence of peroneal nerve in mice lacking RhoE expression

**Figure 5:**
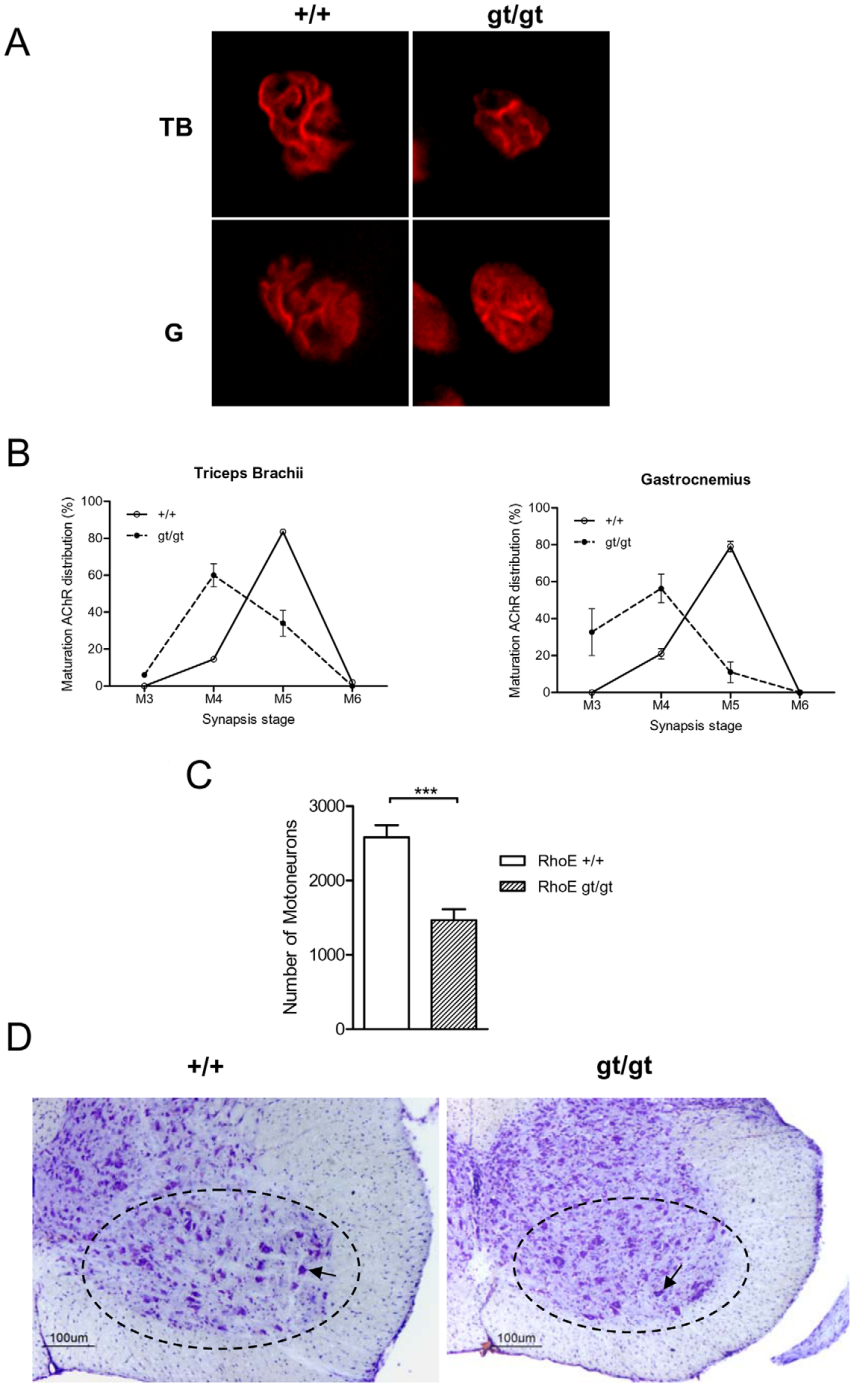
RhoE gt/gt mice show a delay in neuromuscular junction maturation and a decrease in the number of spinal motoneurons. **A**) Representative synaptic AChR cluster morphologies in the neuromuscular junctions of wild type (left) and RhoE gt/gt (right) mice at postnatal day 21, both in the triceps brachii (TB, top) and in the gastrocnemius (G, bottom) muscles. The picture of the RhoE gt/gt mouse corresponds to the M4 stage of neuromuscular junction, whereas the image of the wild type shows a more developed M5 form of synaptic cluster. **B**) Type of synaptic AChR clusters in PD21 RhoE gt/gt and wild type mice according to the status of maturation in the triceps brachii (left) and gastrocnemius (right) muscles. The forms of synaptic clusters that are present in the RhoE gt/gt mice are less developed than in the wild types. **C**) Motoneurons from 4 RhoE wt and 5 RhoE gt/gt mice were counted as described in Materials and Methods. RhoE gt/gt mice show a significantly reduced number of motoneurons (1467±146 vs 2582±322, p<0.001 in a Student's *t* test). **D**) Representative pictures of RhoE +/+ and RhoE gt/gt ventral horns (dotted lines) of cervical spinal cord sections. Arrows point at one motoneuron in each section.

The poor performance in the motor tests along with the abnormal posture of the RhoE gt/gt mice, led us to investigate whether these alterations could have a structural basis. We therefore analyzed the structure of the muscle fibers. Histological analysis of mutant muscle fibers did not show any apparent anomaly nor any increase in the number of central nuclei, which would suggest muscular degeneration or regeneration. Nerve cross sections did not show any gross alteration either (data not shown). However, neuromuscular synapses were less developed in RhoE gt/gt mice than in their control littermates (**Figure 5A**). We specifically found a delay in neuromuscular junction maturation, with an increased presence of less developed plates, both in the forelimbs (triceps brachii) and in the hindlimbs (gastrocnemius), at PD21 (**Figure 5B**). We finally studied whether the number of motoneurons was affected in the ventral spinal cord of RhoE gt/gt mice and found a reduction of 43% (p<0.001) in the number of motoneurons when compared to the +/+ mice (**Figure 5C and 5D**).

**Figure 6:**
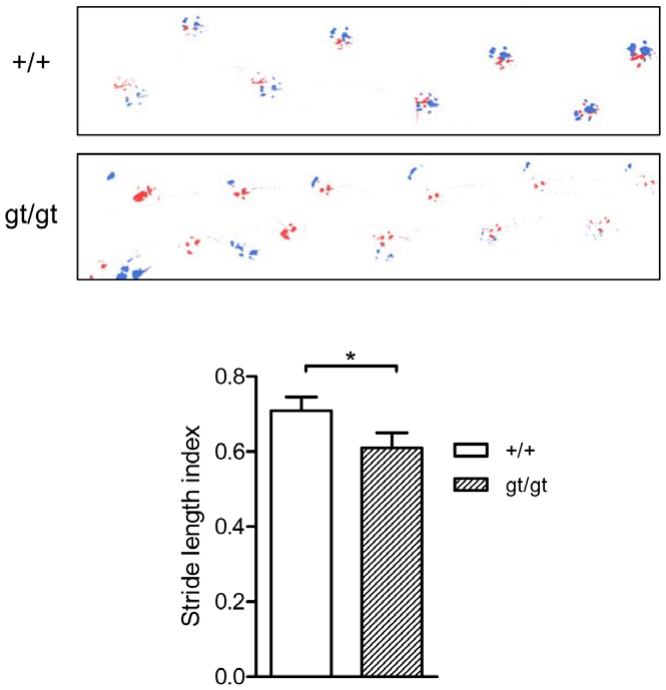
Abnormal walking of RhoE gt/gt mice. Representative walking footprint patterns of PD21 wild type (top) and RhoE gt/gt (bottom) mice. Forepaws were stained in red and hindpaws in blue. The pattern clearly differs, showing shorter and irregularly spaced strides in RhoE gt/gt mice when compared to the wild types. The graph shows the quantification of the stride length index of wild type and RhoE gt/gt mice. Since RhoE gt/gt mice were smaller than the wild types, the stride length index was calculated as the ratio stride-length/body-length for each mouse. The stride length index was reduced in the RhoE gt/gt mice compared to the control mice (*p<0.05 in a Student's *t* test).

**Figure 7:**
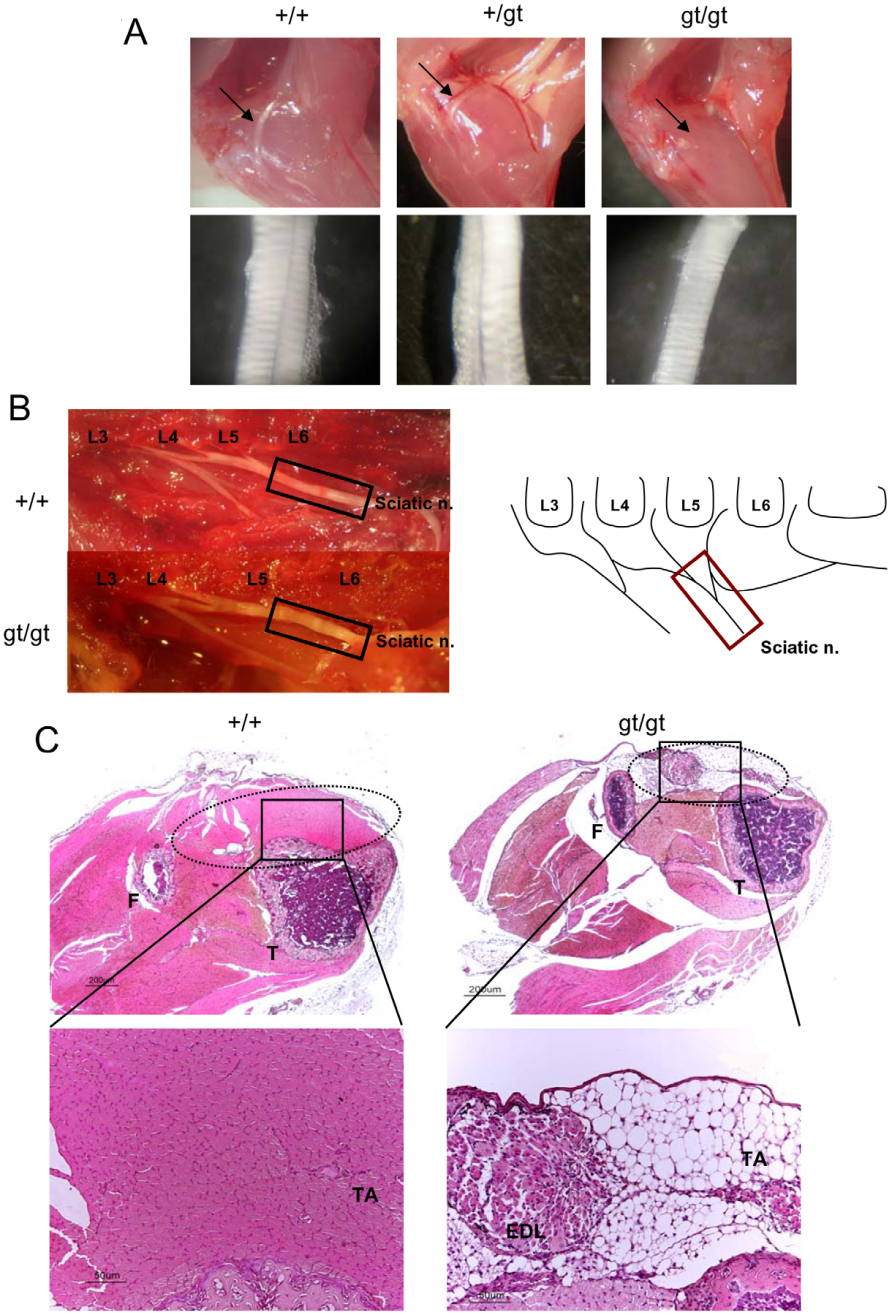
Absence of the common peroneal nerve and disappearance of the craniolateral hindlimb muscle fibers in RhoE gt/gt mice. **A**) Top panels: Dissection of the hindlimb of representative PD21 wild type, RhoE +/gt and RhoE gt/gt mice. The common peroneal nerve was absent in all the RhoE gt/gt mice (right panel). Arrows indicate the presence of the common peroneal nerve in the wild type (left panel) and in the heterozygous (central panel) and its absence in the RhoE gt/gt sample (right panel). Botom panels: The two main components (tibial and common peroneal) of a sciatic nerve removed from a wild type mouse (left panel) and form a heterozygous central panel) can be observed, whereas the sciatic nerve of a RhoE gt/gt mouse only shows the tibial nerve. **B**) The spinal roots originating the sciatic nerve in a wild type (top) and in a RhoE gt/gt (bottom) mouse are similar. The figure on the right shows a schematic representation of the roots. **C**) Histological section of a left P15 hindlimb from a wild type (left panels) and a RhoE gt/gt (right panels) mouse. The lower panels show a higher magnification of the area in the upper rectangle. The dotted lines represent the area covered by the craniolateral muscles in the wild type and in the RhoE gt/gt mice. Note that in the RhoE gt/gt hindlimb almost all muscle cells have been replaced by adipose tissue and clusters of small size fibers. TA: tibialis anterior muscle, EDL: extensor digitorum longus muscle, T: tibia, F: fibula.

One of the most striking findings was that all RhoE gt/gt mice displayed an abnormal hindlimb position characterized by hyperextension of the feet and impossibility to flex them (see **Figure 2A** and **Video S1**). Accordingly, the behavioral tests indicated worse performances in the hindlimbs grasping and placing tests. In addition, as it can be noted in the footprint analysis, RhoE gt/gt mice hindlimb steps were shorter and irregular and showed a reduced stride length (15%, p<0.05) when compared to their wild type littermates (**Figure 6**). In order to analyze the anatomical integrity of the hindlimbs, we dissected the limbs of RhoE gt/gt mice. Our analysis showed, in all the animals analyzed (n = 37), a complete absence of the common peroneal nerve (**Figure 7A**, top panels). Consequently, the mutant sciatic nerve, instead of splitting into the common peroneal and the tibial nerves, was only continued by its tibial component (**Figure 7A**, bottom panels). The sciatic nerve of RhoE +/gt mice were similar to the wild types (**Figure 7A**). We also found that all the spinal roots which anatomically originate the sciatic nerve were present in the mutant mice (**Figure 7B**). The absence of the peroneal nerve in RhoE gt/gt mice resulted in the atrophy of its target muscles, which are normally located in the craniolateral compartment of the distal hindlimb (**Figure 7C**). These muscles in the RhoE gt/gt mice appeared much reduced in size, with most of the muscle fibers replaced by adipose and connective tissue, whereas the remaining muscle cells appeared grouped in clusters of small size fibers (**Figure 7C**). All other main muscles and nerves of both the fore- and the hind-limbs appeared normally located although the muscles of RhoE gt/gt mice were reduced in size and volume when compared to the control littermates.

## Discussion

Rnd proteins are atypical members of the Rho family and their functions *in vivo* have been less studied. The recent reports showing that Rnd2 is essential for the migration of radial cortical neurons acting downstream of neurogenin2 [18], and that Rnd1 and Rnd3 are required for *Xenopus* somitogenesis [20] suggest that Rnd proteins could be important during development. However, the generation of mice lacking Rnd proteins, which would provide new tools to study the *in vivo* function of these proteins, has not yet been described. Here, we analyze the phenotype of RhoE null mice and show that RhoE is essential for a correct development since the absence of RhoE expression induces severe behavioral and structural alterations and becomes deleterious few weeks after birth. Our results show that RhoE has unexpected functions during the development since mice lacking RhoE expression have neuromotor impairment and neuromuscular alterations. Surprisingly, RhoE deficiency also results in the complete absence of the common peroneal nerve. As RhoE is highly expressed not only during postnatal but also embryonic development, the absence of RhoE expression during prenatal development is also very likely to contribute to the phenotypes observed in RhoE null mice. This is supported by the fact that most of the deficits observed in RhoE null mice are already present at birth. The real impact of the absence of RhoE expression during the embryonic development will be addressed in future studies.

The ablation of RhoE expression leads to a delayed maturation of several systems, especially of those that affect motor development, as it can be concluded from the results of the motor tests, which are notably worse performed by RhoE null mice. The neuromotor development is a complex process that has to be interpreted globally. In our model, the time-dependent changes in pivoting activity combined with the delayed latency observed in the walking activity, reflect a delay in neuromotor development. In fact pivoting activity is an immature motor pattern that disappears along time, (as can be seen in **Figure 4A** for wild type mice). This is accompanied with a progressive decrease in the time needed to initiate the straight line walking (**Figure 4D**) as the cranio-caudal maturation is completed. Instead, RhoE gt/gt mice were hypoactive in early time points (PD5–7), with less pivotings and longer latency to walk than their siblings, and showed a delayed neuromotor development characterized by the persistence of the immature motor pattern in the pivoting test and a delay in the appearance of a normal gait pattern. Moreover, the worse performance of the RhoE gt/gt mice in a broad range of motor tests indicates that RhoE could be involved in the correct development of the motor function. These results suggest that RhoE is involved in the maturation of the neuromotor system at different levels. Some of these defects could have been originated in different encephalic structures. For example, the limb clasping phenotype along with ataxia can have a cerebellar origin [21]. RhoE expression in the cerebellum is restricted to the EGL. Although we have not observed gross alterations of the cerebellum in RhoE gt/gt mice, we cannot rule out that molecular changes in these cells in the RhoE null mice could be responsible for some of the effects. As RhoE is widely expressed in the central nervous system, as we have previously shown [14], other encephalic structures could also be altered in RhoE null mice. Therefore, a careful analysis of RhoE gt/gt mice brains will be performed.

It is interesting to note that many Rho GTPases regulate axon outgrowth and growth cone formation and/or collapse (reviewed in [22]). Additionally, we have recently shown that RhoE stimulates neurite-like outgrowth in PC12 cells through the inhibition of RhoA/ROCKI signaling [15]. Therefore, the delayed maturation observed in many neuronal functions of RhoE null mice could be related to neurite outgrowth retardation. The less developed stage of the motor endplates that we have observed in the RhoE gt/gt mice could also be due to similar causes. Nevertheless, the actual state of neurite outgrowth of RhoE gt/gt neurons is currently under study.

The lack of RhoE expression results in a decrease in muscle mass and in the number of motoneurons, and in a delay of the neuromuscular junction maturation. Interestingly, recent evidences show that RhoE expression is involved in muscle maturation. Specifically, RhoE is essential for myoblast elongation and alignment before fusion, although it is dispensable for myogenesis induction [23]. RhoE is also upregulated in C2C12 cells overexpressing Gαz, resulting in the inhibition of myogenic differentiation [24]. In addition, RhoE has recently been shown to interact with PLEKHG5/Syx [25], a brain-specific RhoA GEF (guanine exchange factor, a positive regulator of Rho GTPases) [26]. This protein has been found to be mutated in a form of Lower Motor Neuron Disease (LMND), characterized by childhood onset and generalized muscle weakness and atrophy with loss of walking [27]. Moreover, other Rho GTPases such as RhoA and Rac1 have also been demonstrated to be involved in motoneuron survival, and mutations of ALS2, which encodes alsin, a GEF with dual specificity for Rac1 and Rab GTPases, cause a form of juvenile-onset of the degenerative disorder of motor function Amyotrophic Lateral Sclerosis (reviewed in [17]). Our findings confirm that RhoE is highly expressed in the forming striated muscles as well as in the central nervous system (this work and reference [14]).Thus, we can speculate that the absence of RhoE expression could result in a delay of myogenesis that would produce a reduction of muscle mass which, in turn, would decrease the number of motoneurons as a consequence of a lesser production of trophic factors . On the other hand, the smaller number of motoneurons found in the RhoE gt/gt mice could be a direct consequence of the lack of RhoE expression and this, in turn, would result in the reduction of muscular mass observed in these animals. All these data suggest that RhoE could be involved in motoneuron diseases and that the RhoE mutant mouse presented in this work could be a useful animal model for these diseases.

One of the most intriguing observations of this work is that the lack of RhoE expression results in the absence of the common peroneal nerve, accompanied by the atrophy of its target muscles which are located in the craniolateral compartment of the distal hindlimb. The abnormal hindlimb position and the delayed appearance of the hindlimb grasping and placing reflexes in RhoE gt/gt mice is likely to be a consequence of such nerve absence. Strikingly, in addition to RhoE, other apparently unrelated genes as the ephrin receptor EphA4 [28] and the HoxD locus [29] have been reported to be responsible for the proper development of the peroneal nerve. Similarly to RhoE, EphA4 ablation induces the absence of the peroneal nerve and muscular atrophy of its target muscles, but with slightly lower penetrance (88% of hindlimb affected in EphA4 −/− *vs* 100% in RhoE gt/gt). In contrast, whereas about 30% of EphA4 heterozygotes display such anomaly, none of the RhoE +/gt mice was affected.

Emerging evidence shows that activation of Eph-mediated downstream signaling involves Rho proteins leading to actin cytoskeleton reorganization. Several studies have identified signaling molecules downstream of Eph activation, the majority of which converge to the regulation of small Rho GTPases including Rac1, Cdc42, and RhoA. For example, activation of EphA by its ligand leads to a transient inhibition of Rac1 activity, concomitant with RhoA activation [30], [31]. In addition, various GEFs have been identified as intermediaries that link EphA receptors to small Rho GTPases at growth cones [31], [32], [33]. Finally, GTPase-activating proteins (GAPs), which act as negative regulators of Rho GTPases are also involved in Eph signaling [34], [35]. To date, no direct relationship has been described between EphA4 and RhoE, but the similarity of the phenotypes observed in EphA4 −/− and RhoE gt/gt, along with the Eph-RhoA relationship and the RhoA-RhoE antagonism, suggest that RhoE could be involved in the EphA4 signaling pathway. In this context, it is noteworthy that it has been recently found a novel interaction between EphA1 and the Integrin-Linked Kinase (ILK) [36], a mediator of interactions between integrin and the actin cytoskeleton that regulates RhoE to control Schwann cell process extension [37].

Loss of HoxD10 function, alone [29], [38] or together with Hoxc10 [39], also results in the absence of the peroneal nerve, probably because of the disorganized pattern of the nerve formation. Since no relationship has been established between the *hox* genes and Rho proteins to date, it would be interesting to analyze whether RhoE is altered in the HoxD mutant mice, as well as in the EphA4 −/− mice.

How the absence of RhoE produces all these anomalies is currently under research. We have analyzed the protein expression profile in extracts from RhoE gt/gt and wild type brains and the result shows that the highest difference is in cofilin (manuscript in preparation). Cofilin is an actin severing protein involved in actin depolymerization and therefore in several events as cell motility, growth cone collapse and axon repulsion (reviewed in [40]). Cofilin activity is regulated by phosphorylation/dephosphorylation events [41]. The Rho/ROCK pathway can regulate cofilin phosphorylation through LIMK [42]. We have recently demonstrated that RhoE induces neurite outgrowth in PC12 cells through inhibition of the RhoA/ROCK pathway resulting in dephosphorylation of cofilin [15]. All these data would suggest that the absence of RhoE expression in RhoEgt/gt mice would result in the activation of the RhoA/ROCK pathway that in turn would inactivate cofilin, resulting in neurite extension and/or cell migration alterations.

In summary, the absence of RhoE expression results in a phenotype characterized by the abnormal development of the nervous system, reduced body size and lethality few weeks after birth. Therefore, with our *in vivo* system, RhoE reveals itself as a very important protein for the normal development of vertebrates. Moreover, our results suggest that RhoE (and/or its signaling pathway) could be involved in neurological disorders as many other members of the Rho family [43]. Our recent reports showing high levels of RhoE expression in widespread areas of the central nervous system, including the motoneurons [14], and the role of RhoE as a promoter of neurite formation [15] could help to explain the importance of this protein in the nervous system and the alterations observed in the mice lacking RhoE expression.

## Materials and Methods

### Ethics Statement

All animal procedures were approved by the local ethics committees (Ethics Committee for Animal Research of the Barcelona Biomedical Research Park, ID#MDS-08-1060, and Ethics Committee for Animal Welfare of the Universidad CEU Cardenal Herrera, ID#CEBA03/2007), met the local guidelines (Spanish law 32/2007), European regulations (EU directive 86/609) and Standards for Use of Laboratory Animals nu A5388-01 (NIH). The experimenters hold the official accreditation for animal work (Spanish law 32/2007).

### Generation of RhoE gt/gt mice

Mice deficient for RhoE expression were generated at Lexicon Pharmaceuticals, by gene trapping in ES cells, identification of trapped genes by using OmniBank™ Sequence Tags (OSTs) and characterization of retroviral gene-trap vector insertion points as previously described [19], [44]. OmniBank ES cell clone OST364657 was used to generate RhoE gt/gt mice as described [44]. The gene-trap vector VICTR 37 was inserted within intron 2 of the *RhoE* gene. All mice were of mixed genetic background (129SvEvBrd and C57Bl/6J). Mice were genotyped by PCR. The wild-type locus yields a PCR product of 600 bp using primers 5′-TTT ACA CAG TAG GCT GAC TC-3′ and 5′-TGA GCT AGG AAG ATG CGG ATG T-3′. The mutant locus yields a PCR product of 400 bp using primers 5′-AAA TGG CGT TAC TTA AGC TAG CTA GCT TGC-3′ and 5′-TGA GCT AGG AAG ATG CGG ATG T-3′ (**Figure 1B**). PCR products were separated through 1% agarose electrophoresis gels.

The expression of RhoE was analyzed by Western Blot of extracts from Mouse Embryonic Fibroblasts (MEFs) of the three genotypes. Cell extracts were prepared as described [12]. We used an anti-RhoE monoclonal antibody (clone3, Upstate, Lake Placid, NY, USA) and horseradish peroxidase-conjugated secondary antibodies (Pierce, Rock-Ford, IL, USA). Blots were developed using the enhanced chemiluminescence system (ECL Plus, Amersham Bioscience, Little Chalfont, UK).

Same sex littermates were group-housed (4–6 animals per cage) under a 12-h light/dark schedule in controlled environmental conditions of humidity and temperature with food and water supplied *ad libitum*. As RhoE gt/gt mice died shortly after weaning, they were kept with their mother until they died or were sacrificed when their condition worsened.

### Somatometric and postnatal neurobehavioral analysis

Mice used for behavioral analysis were derived from crosses between RhoE +/gt animals. The day of birth was considered PD1. All the behavioral tests were conducted by the same experimenter in an isolated room and at the same time of the day. The experimenter was blind to genotype. A total of 49 mice (11 RhoE gt/gt, 30 RhoE +/gt and 8 RhoE +/+) from six different litters were examined during the pre-weaning period. Somatometry was performed daily (PD1 to PD21) by weighing the pups and measuring the body length. The day of appearance of developmental landmarks was recorded and used as the unit of analysis. Neurobehavioral analysis included a battery of tests evaluating pre-weaning sensorial and motor responses that reflect the maturation of the CNS [45]. The following tests were performed:

#### Crossed extensor reflex

Pinching the foot of one hind limb causes flexion of the stimulated limb, while the opposite hind limb is extended.

#### Rooting reflex

Bilateral stimulation of the face region stimulates the animal to crawl forwards pushing the head in a rooting fashion.

The day in which these two reflexes were lost was recorded.

#### Righting Reflex

When the animal is placed on its side, it immediately turns over to rest in the normal position with all four feet on the ground. The first day when the animal is able to turn over in less than 30 seconds was recorded.

#### Fore and hind limb placing responses

Contact of the dorsum of the foot against the edge of an object will cause the foot to be raised and placed on the surface of such object when the animal is suspended by the tail.

#### Grasp reflex

When the fore or hind foot is stroked with a blunt instrument, it is flexed to grasp the instrument.

#### Vibrissae placing response

When the mouse is suspended by the tail and lowered so that the vibrissae make contact with a solid object, the head is raised and the fore limbs are extended to grasp the object.

The first day in which the placing and grasping reflexes appeared was recorded.

#### Preyer's response

A loud, sharp noise causes an immediate startle response, observed as a sudden extension of the head and fore and hind limbs which are then withdrawn and a crouching position is assumed.

#### Tactile startle response (blast response)

A strong blow causes an immediate startle response, a sudden extension of the head and fore and hindlimbs, which are then withdrawn and a crouching position assumed.

The first day when the normal startle responses were observed was used for comparison between the three genotypes.

#### Gait analysis and developmental locomotor patterns

Pivoting is an immature locomotion characterised by rotations around the hindlimbs and is due to the later development of these hindlimbs. The ability to walk in a straight line is an indicative of a more mature development. At different ages, the number of pivotings (measured as 90° turns) in 60 seconds was recorded. The latency to walk was measured as the time the mouse waits until it starts moving in a straight line for a distance equal or higher than its own length in less than 60 seconds.

#### Wire suspension test

The ability to hold on to a 4 mm bar for a given amount of time was measured, as latency to fall. The maximum time allowed was 60 seconds.

#### Negative geotaxis

When the mouse is placed on a 45° angle slope with its head pointing down the incline, it will turn around and crawl up the slope. The day when the animal was able to turn around in less than 35 seconds was recorded.

#### Vertical Clinging and Climbing

The pup is held against a vertical metallic grid (wire: 0.6 mm in diameter, mesh: 6 mm wide). Two behaviours are scored: clinging for 10 s and climbing after clinging. The first day when the test is positive was recorded.

The limb clasping test consists in analyzing the response of the mouse limbs when the mice are held suspended in the air by the tail. The wild type mice open their limbs widely [46], [47].

### Footprint test

The hind- and forepaws of the mice were coated with blue and red nontoxic paints, respectively. The animals were then allowed to walk along a 50 cm-long, 10 cm-wide runway into an enclosed box (with 10 cm-high walls). All mice had two training runs. The footprint patterns were analyzed for three step parameters (measured in cm): (1) Stride length was measured as the average distance of forward movement between each stride, (2) hind-base width and (3) front-base width were measured as the average distance between left and right hind footprints and left and right front footprints, respectively. These values were determined by measuring the perpendicular distance of a given step to a line connecting its opposite preceding and proceeding steps [48].

### X-Gal staining

Embryos from RhoE +/gt mice crosses were obtained by cervical dislocation or by overdose of pentobarbital of the mother at 14.5 days post coitum (dpc). They were then washed in cold-phosphate-buffered saline (PBS) and fixed (2% paraformaldehyde 2 mM MgCl_2_) for 2–4 hours at room temperature, cryoprotected and frozen. Samples were then serially sectioned in a cryostate, postfixed with 0,2% PFA 2 mM MgCl_2_ and permeabilized with detergent solution (0,02% NP40, 0.01% deoxycolate, 2 mM MgCl_2_, in 100 mM sodium phosphate buffer pH 7.3). For X-Gal staining, the embryos were incubated in the dark at 37°C overnight in X-Gal solution (1 mg/ml X-Gal, 5 mM potassium ferricyanide and 5 mM potassium ferrocyanide, 0,02% NP40, 0.01% deoxycolate, 2 mM MgCl_2_, in 100 mM sodium phosphate buffer pH 7.3). Finally, sections were mounted and observed under light microscopy.

### Histology and number of motoneurons

Mice at PD21 (4 RhoE +/+ and 5 RhoE gt/gt) were sacrificed with an overdose of sodium pentobarbital and perfused transcardially with 50 ml of heparinized physiological saline followed by 200 ml of fixative solution containing 4% paraformaldehyde in 0.1 M phosphate buffer, pH 7.4, containing 0.2% picric acid at room temperature. The spinal cord was removed and immersed in 4% paraformaldehyde in 0.1 M phosphate buffer, pH 7.4, for 3 days at 4°C. The spinal cord was cryoprotected in 20% phosphate-buffered sucrose solution. Serial 16 µm thick coronal sections were cut with a cryostat and stained with 1% cresyl fast violet solution. Sections were analyzed with an Olympus BX40 microscope. Spinal motoneurons, identified by their location at the ventral horns as big size cells with a well defined nucleus, were counted by an experimenter blind to genotype. From each animal, a total of 120 sections (40 from each of the spinal regions, cervical, thoracic and lumbar) separated at least 64 µm, were analyzed. Muscle histology was analyzed by immersing the hindlimbs of PD15 mice in formalin for 7 days at room temperature. Then, the limbs were rinsed in water and decalcified with equal parts of 8% hydrochloric acid solution and 8% formic acid solution for 1 day. Once the decalcification was completed, the limbs were rinsed in water and transferred to an ammonia solution for 30 min. After fixation, decalcification and paraffin-embedding, 8 µm thick sections were obtained. The sections were dehydrated with a graded series of increasing ethanol concentrations and stained with hematoxylin-eosin. Cerebellar sections (4 µm thick) were obtained from paraffin embedded PD15 mice brains. Sections were deparaffined and hydrated and stained with cresyl violet solution.

### Immunohistochemistry

Embryos were immersed in 4% paraformaldehyde overnight, dehydrated in increasing concentrations of ethanol, embedded in paraffin, serially sectioned (5 µm) in an HM 310 Microm microtome (Walldorf, Germany) and collected on polylysine-coated slides. Antigen retrieval of deparaffined and rehydrated sections was performed by heating at 100°C in a water-bath for 20 min in citrate buffer (10 mM pH 8). Sections were then washed three times in 0.1 M phosphate buffer with 0.2% Triton X-100 (PBST) and incubated with 3% H_2_O_2_ in methanol for 40 min to quench endogenous peroxidase activity. Nonspecific binding was blocked with 10% horse serum in 3% BSA. Immunohistochemistry for RhoE (Upstate, Lake Placid, NY, USA, 1∶50) was performed using the immunoperoxidase procedure of Vectastain Elite ABC kit (Vector Laboratories, Burlingame, CA, USA). Briefly, the sections were incubated with the primary antibody overnight at 4°C in a humidified chamber. They were then incubated 1 h with a biotinylated secondary anti-mouse antibody, amplified with the avidin–peroxidase complex and finally revealed by diaminobenzidine tetrahydrochloride stain.

### Morphological analysis of neuromuscular junctions

Mice were sacrificed as described above and the limbs were dissected to expose the triceps brachii and gastrocnemius muscles. Muscles were fixed in 4% PFA, sunk in 30% sucrose, frozen and sectioned in a cryostat. Sections were then stained with alpha-bungarotoxin-tetramethylrhodamine and visualized with fluorescence microscopy. The process of maturation of the Acetilcholine (AChR) clusters distribution at the neuromuscular junctions (NMJs) was classified into six stages (M1 to M6), as previously described [49].

### Data analysis

As no significant differences were detected between male and female mice of each genotype, results were combined. Unless stated otherwise, the significance of the effects was assessed by a one-way or multivariate analysis of the variance (ANOVA) with Bonferroni's post hoc analysis test. All analyses were performed by using the statistical package GraphPad Prism (5.0).

## Supporting Information

Video S1 **RhoE gt/gt mice are smaller and show an abnormal hindlimb position.** Recording showing one PD7 RhoE gt/gt mouse (left on the screen) with one wild type littermate. The RhoE gt/gt mouse shows an abnormal position of the hindlimbs due to the absence of the common peroneal nerve, with the characteristic foot drop and narrowing of the toe spread, and uncoordinated movements. Note that the RhoE gt/gt mouse is much smaller that the wild type.
(WMV)
